# Effects of Whey Protein Supplement on 4-Week Resistance Exercise-Induced Improvements in Muscle Mass and Isokinetic Muscular Function under Dietary Control

**DOI:** 10.3390/nu15041003

**Published:** 2023-02-16

**Authors:** Chae-Been Kim, Jong-Hoon Park, Hyoung-Su Park, Hye-Jin Kim, Jung-Jun Park

**Affiliations:** 1 Department of Sport Science, Pusan National University, Busan 46241, Republic of Korea; 2 R&D Group, Maeil Health Nutrition Co., Ltd., Pyeongtaek 17714, Republic of Korea

**Keywords:** whey protein supplementation, muscle mass, isokinetic muscular function, dietary control

## Abstract

(1) The purpose of this study was to investigate the effect of whey protein supplementation under dietary control on improvements in muscle mass and function following resistance exercise training. (2) Thirty-two men were randomly assigned to a whey protein supplementation group taking whey protein isolate (PSG, *n* = 17) and a placebo group (CON, *n* = 15). Participants were provided with three meals per day corresponding to the estimated individual daily energy intake. The supervised resistance exercise program was conducted 60 min per day, six days per week, for four weeks. (3) Post-intervention, there was a significant interaction between groups in terms of muscle mass increase (*p* = 0.033, η^2^ = 0.14), with a greater increase in the PSG. There were also significant interactions between the groups and increases in peak torque of the dominant knee flexors (*p* = 0.048, η^2^ = 0.12), dominant shoulder extensors, and non-dominant shoulder extensors (*p* = 0.028, η^2^ = 0.15; *p* = 0.015, η^2^ = 0.18), and the total work of the dominant knee and shoulder extensors (*p* = 0.012, η^2^ = 0.19; *p* = 0.013, η^2^ = 0.19), with greater increases in the PSG. (4) These results suggest that whey protein supplementation enhances resistance exercise-induced increase in muscle mass and overall muscular strength and endurance, independent of dietary influence.

## 1. Introduction

The popularity of dietary supplements has steadily increased. Similarly, in the sports field, professional athletes and the general public who enjoy leisure activities consume a variety of health supplements. In particular, many people take protein supplements to improve muscle hypertrophy and function in combination with resistance exercise [1,2].

Among dietary supplements, carbohydrate supplementation is the main source of energy for muscle contraction, whereas amino acid supplementation not only provides energy but also affects muscle protein synthesis and muscle recovery after resistance exercise [3]. Protein supplements with an optimal amino acid ratio can be useful for muscle protein synthesis and recovery for people who perform resistance exercises [4]. Whey protein is considered the gold standard of protein supplements [5] because it contains a higher essential amino acid content than other protein supplements [6]. In addition, the amino acid component of whey protein has a pattern similar to that of human skeletal muscle amino acids; therefore, it is absorbed faster than other protein sources [7].

Many studies have investigated the effect of whey protein supplement intake on the increase in muscle mass and strength associated with resistance exercise. However, the results of these studies have been inconsistent. Several review papers [8,9,10,11,12] and meta-analyses [13,14,15,16,17,18] have reported conflicting results regarding the effects of protein supplementation on resistance exercise-induced improvements in muscle mass and strength. This discrepancy may be due to a variety of factors, including differences in age, training experience, and the amount, type, and duration of protein supplementation in most previous studies. Another important reason for this discrepancy could be the amount of protein that participants consumed from their normal diet during the study period. This is because it can alter the effects of protein supplementation on muscle mass and function during resistance exercise. Nevertheless, few studies have investigated the effects of whey protein supplementation under dietary control.

Most studies on the effects of whey protein supplementation on resistance exercise, even when the normal diet is controlled, have been conducted using food diaries or questionnaires reported by the participants on their diet records [1]. However, if the diet is controlled with a diet log or questionnaire, dependence on the study participant is high, and the longer the intervention period, the more inaccurate the study participant’s record [19]. Additionally, dietary control may not be clear because the economic situation or willingness to participate in the study may change [20]. Therefore, more direct dietary interventions are needed to investigate the effect of whey protein supplementation on resistance exercise-induced improvements in muscle mass and function.

In this study, we investigated the effect of whey protein supplementation on changes in muscle mass and isokinetic muscle function following resistance exercise while controlling the participants’ normal diet by providing three meals a day during the entire study period.

## 2. Materials and Methods

### 2.1. Participants

Thirty-six men in their 20s and 30s living in metropolitan B in South Korea were recruited for this study. We recruited men with standard Korean body sizes (height: 174 ± 5 cm, weight: 74 ± 4 kg) for the study [21] in order to reduce the variation in physique and sex differences as much as possible. All participants were non-smokers and relatively healthy sedentary individuals (no exercise within 3 months). They also had no allergies to dairy or lactose intolerance and no alcohol consumption within the last 3 months. They were randomly assigned to a whey protein supplement intake group (PSG) and a placebo intake group (CON) (18 participants in each group). However, mainly due to COVID-19, 1 PSG participant, and 3 CON participants dropped out; therefore, only the results of 32 (PSG, *n* = 17; CON, *n* = 15) participants were finally included in the data analysis. This study was approved by the Institutional Review Board of Pusan National University (No. 2020_136_HR, date of approval: 31/05/2021), and all participants signed an approved consent form before participating in the study. This study was conducted in accordance with the Declaration of Helsinki of 1975, revised in 2013.

### 2.2. Study Design

This study was designed as a randomized, double-blind, placebo-controlled trial. After the random assignment of participants into 2 groups, the PSG was given a whey protein supplement, and the CON was given a placebo containing carbohydrates. Both groups underwent resistance exercise training for 4 weeks. For dietary control, each participant was given 3 meals a day corresponding to the estimated daily energy intake calculated by the individual amount of exercise and resting metabolic rate for the entire period of the study. Body composition and isokinetic muscular function were measured before and after the intervention.

### 2.3. Dietary Control

The daily calorie intake for each participant was calculated by multiplying the individual’s resting metabolic rate (RMR) by the activity factor [22]. All participants were provided 3 meals a day, which was equal to their calculated daily calorie intake during the 4 weeks of intervention. A packed meal company (Salady Inc., Seoul, Republic of Korea) prepared each meal for each participant at a ratio of 10% protein, 30% fat, and 60% carbohydrates based on the individual’s calculated daily calorie intake. All meals were delivered directly to participants every morning. The nutritional content and calories of the meals provided to the participants were analyzed weekly. Compliance with the diet was monitored through videos sent by the participants to record what they ate at each meal.

### 2.4. Whey Protein Supplement and Placebo

The whey protein supplement and placebo contained the same calorie content of 99 kcal per pack and were administered to the PSG and CON respectively. The whey protein supplement and placebo were formulated in powder form by Maeil Health Nutrition Co. Ltd. (Gyeonggi-do, Republic of Korea). One pack of whey protein powder supplement contained 20 g of whey protein isolate, 8 g of carbohydrate, and 6.4 g of dietary fiber, and one pack of placebo contained 25 g of carbohydrate instead of protein. Each participant consumed either a whey protein supplement or a placebo 3 times a day: morning, post-exercise, and evening. All powders were flavored with chocolate to mask their contents and were provided in unlabeled packaging to ensure that the study participants or researchers could not tell them apart.

### 2.5. Resistance Exercise

All participants performed resistance exercise at 60–70% of 1-repetition maximum (RM) for 60 min per day, 6 days per week for 4 weeks. The exercise program consisted of 5 min of warm-up, 50 min of main exercise, and 5 min of cool-down exercise, and 1 day of rest was taken after 6 days of exercise. The exercise program was divided into chest, back, shoulder, arm, abdomen, and lower-body exercises. The daily exercise program was split into a combination of upper- and lower-body exercises. To gradually increase the intensity, exercise was performed at 60% of the 1-RM in weeks 1 and 2 and 70% of the 1-RM in weeks 3 and 4. All participants were advised to refrain from any additional exercises other than the exercise program conducted in this study.

### 2.6. Measurements

#### 2.6.1. Body Composition

To minimize the measurement error, food, beverage, alcohol, and caffeine intake was restricted 8 h before the measurements. Each participant underwent measurements 3 times over 3 separate days using a bioelectrical impedance analyzer (Inbody 620, Inbody, Republic of Korea). All measurements were conducted simultaneously in the morning, and the average values were used for statistical analysis.

#### 2.6.2. Resting Metabolic Rate

The RMR test was performed using a gas analyzer (Quark b^2^ RMR, COSMED, Italy). All tests were conducted after fasting for at least 8 h and resting for 10–15 min after arriving at the laboratory. A canopy was placed over the participant’s head, and the participant’s breathing gas was measured for 15 min while lying on the bed. The first 5-min gas measurements were eliminated, and the remaining 10-min gas measurements were used to determine the RMR. During the test, all participants were instructed not to fall asleep in order to collect accurate data.

#### 2.6.3. Isokinetic Muscular Function

Muscular strength and endurance were measured using an isokinetic dynamometer (Cybex 770, Humac Norm, Stoughton, MA, USA). The flexor and extensor muscles of the knee, shoulder, and trunk were measured to evaluate muscular strength and endurance. Both the dominant and non-dominant knees and shoulders were measured. Muscular strength was evaluated using peak torque at 60°/s with 5 repetitions. The peak torque is determined as the highest torque at any point within any repetition and range of motion and is expressed in N·m. Muscular endurance was evaluated by total work at 180°/s with 15 repetitions. The total work is determined by the sum of the work performed in each repetition and is expressed in Joules (J), which is the total area under the torque. There were 3 practice repetitions before testing to familiarize the participants with the test. The range of motion for the knee was set at 0–90°, the shoulder at 0–135°, and the trunk at −15–95°.

#### 2.6.4. 1-RM Test

The 1-RM test was conducted to determine the intensity of resistance exercise. To reduce muscle fatigue, the rest time between the test set was 3–5 min, and each participant was instructed to perform a sufficient warm-up before the test. The test weight was set to a weight that could be performed for less than 10 repetitions, and 1-RM was calculated using an estimation formula [23].

$$Predicted\;1-R=\frac{Weight\;lifted}{1.0278-0.278x}$$

### 2.7. Data Analysis

Data were analyzed using SPSS version 27 (OBM Corporation, Armonk, NY, USA). Repeated-measures ANOVA with a mixed design was performed to evaluate the interaction between the groups and the change in variables after the resistance exercise. An independent samples *t*-test was used to evaluate the differences at baseline between the 2 groups. A paired *t*-test was used to evaluate differences within groups after resistance exercise. The statistical significance level was set at *p* < 0.05.

## 3. Results

### 3.1. Participant Characteristics

The average age, weight, height, and body mass index (BMI) of the PSG were 23.5 ± 2.7 years, 72.9 ± 2.4 kg, 174.4 ± 3.1 cm, and 24.0 ± 1.2 kg/m^2^, respectively. The average age, weight, height, and BMI of the CON was 24.5 ± 3.3 years, 73.7 ± 2.4 kg, 174.4 ± 3.5 cm, and 24.3 ± 1.85 kg/m^2^, respectively. The RMR was 1965.0 ± 165.1 kcal in the PSG and 2041.5 ± 210.2 kcal in the CON. There were no significant differences in baseline variables between the two groups.

### 3.2. Calorie Intake

The estimated calorie intake for the PSG and CON was 2358.0 ± 198.1 and 2,449.8 ± 252.3 kcal/day, respectively. The actual calorie intake for the PSG and CON was 2249.6 ± 122.8 and 2322.1 ± 156.2 kcal/day, respectively. There were no differences between the groups in either the estimated or actual calorie intake. However, the actual calorie intake was significantly lower than the estimated caloric intake in the PSG by 108.4 kcal/day (*p* = 0.001) and the CON by 127.8 kcal/day (*p* = 0.020). Nevertheless, there was no difference between the groups for this difference in calorie intake.

### 3.3. Body Composition

The changes in body composition after 4 weeks of resistance exercise are shown in Table 1. Both groups showed significant decreases in body weight, body fat, and body fat percentage. However, there were no significant interactions between the groups or these changes.

### 3.4. Muscle Mass

**Figure 1:**
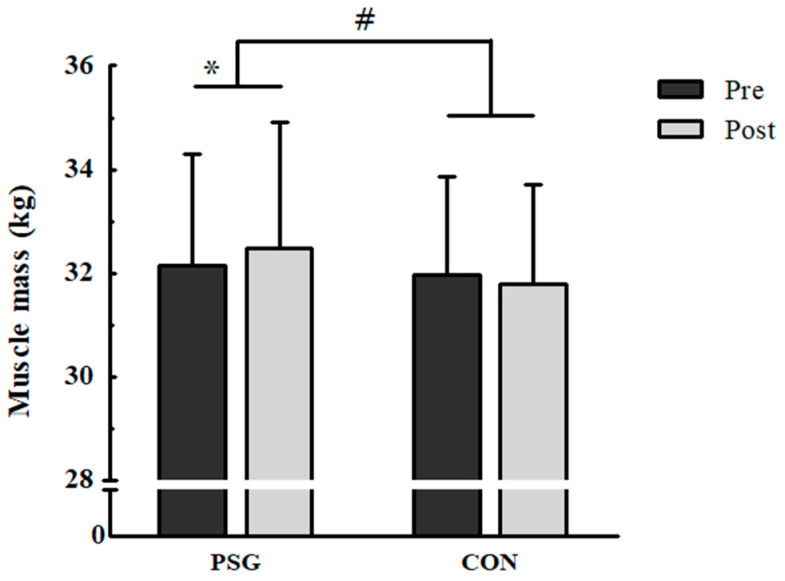
Changes in muscle mass after 4 weeks of resistance exercise. PSG, protein supplement group; CON, placebo group; * Significant difference pre versus post at *p* = 0.033; # significant interaction between the group and the change in muscle mass at *p* = 0.033.

Muscle mass at baseline was 32.14 ± 2.17 kg in the PSG and 31.96 ± 1.91 kg in the CON, and there was no statistical difference between the groups. After 4 weeks of resistance exercise, the PSG showed a significant increase in muscle mass to 32.48 ± 2.43 kg (*p* = 0.033), while the CON did not (Figure 1). There was a significant interaction between the groups and an increase in muscle mass (*p* = 0.033, η^2^ = 0.14).

### 3.5. Isokinetic Muscular Function

**Figure 2:**
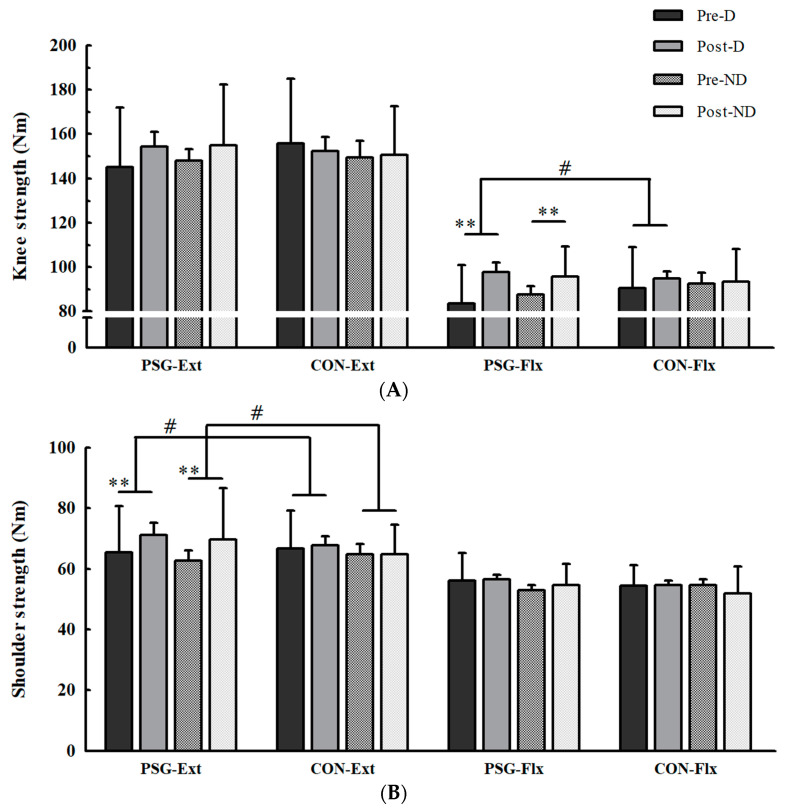
(**A**) Changes in knee peak torque; (**B**) Changes in shoulder peak torque. PSG, protein supplement group; CON, placebo group; Ext, extensors; Flx, flexors; D, dominant; ND, non-dominant; Nm, Newton-meter. Assessments of peak torque consisted of 60°/s during joint flexion-extension. ** Significantly different within the group at *p* < 0.01; # significant interaction between the group and the change in peak torque at *p* < 0.05.

**Figure 3:**
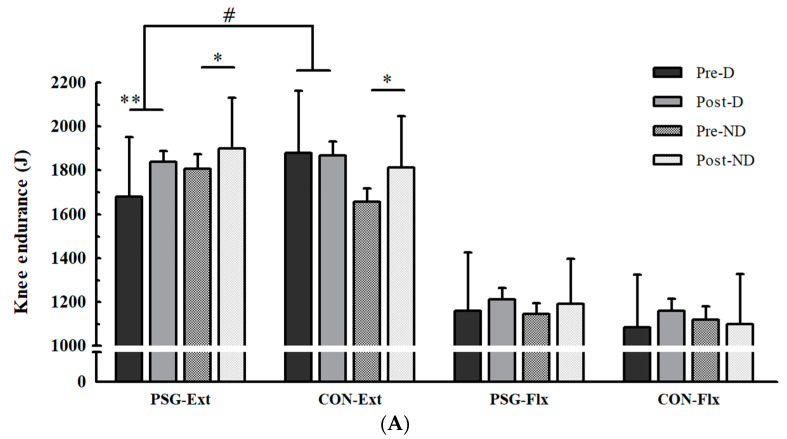

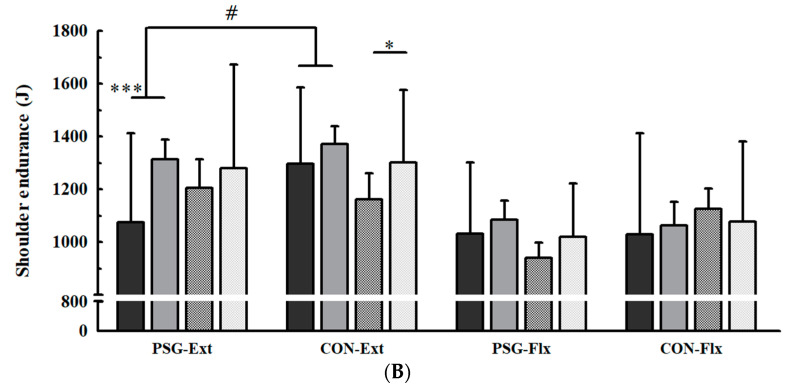
(**A**) Changes in knee joint total work; (**B**) Changes in shoulder joint total work. PSG, protein supplement group; CON, placebo group; Ext, extensors; Flx, flexors; D, dominant; ND, non-dominant joint; J, joule. Assessments of total work were performed at 180°/s during joint flexion-extension. * Significantly different within the group at *p* < 0.05, ** significantly different within the group at *p* < 0.01, *** significantly different within the group at *p* < 0.001; # significant interaction between groups and the change in total work at *p* < 0.05.

The muscular strength and endurance before and after four weeks of resistance exercise are shown in Figure 2 and Figure 3 and Table 2. For muscular strength, there were no baseline differences between the two groups in peak torques of the trunk, dominant and non-dominant knees, and shoulders for extensors and flexors. There were significant interactions between the groups and the change in peak torques of the dominant and non-dominant knees and shoulders (see Appendix A, Table A1, Figure 2). The PSG showed significantly increased peak torques of the dominant knee flexors and dominant shoulder extensors and of non-dominant shoulder extensors, but the CON showed no significant changes. The PSG also showed a significantly increased peak torque of the non-dominant knee flexors, while the CON did not, but there was no interaction. There were no significant changes in the peak torques of the dominant knee extensors, dominant shoulder flexors, non-dominant knee extensors, and non-dominant shoulder flexors in both the PSG and CON. There were also no significant changes in the peak torques of the trunk extensors and flexors in either the PSG or CON (Table 2).

There were no baseline differences in muscular endurance and strength between the two groups in the total work of the trunk, dominant and non-dominant knee, and shoulder for extensors and flexors. There were significant interactions between the groups and changes in the total work of the dominant knee and shoulder (see Appendix A, Table A1, Figure 3). The PSG showed significantly increased total work of dominant knee extensors and dominant shoulder extensors, but the CON showed no significant changes, although the CON showed a tendency to increase the total work of dominant shoulder extensors (*p* = 0.057). There was no significant interaction between the groups and changes in the total work of non-dominant knees and shoulders. However, both the PSG and CON showed significantly increased total work of the non-dominant knee extensors, and the CON showed significantly increased total work of the non-dominant shoulder extensors, while the PSG did not. There were no significant changes in the total work of the dominant knee flexors, dominant shoulder flexors, non-dominant knee flexors, and non-dominant shoulder flexors in both PSG and CON. There were also no significant changes in the total work of the trunk extensors in either the PSG or CON (Table 2). However, the PSG showed significantly increased total work of the trunk flexors, while the CON did not, but there was no interaction.

## 4. Discussion

In this study, we investigated the effects of whey protein supplementation on improvements in muscle mass and isokinetic muscular function, followed by resistance exercise. We designed this study as a double-blind, randomized, placebo-controlled trial, and to eliminate dietary effects, we provided all study participants with three meals a day based on the calculated individual estimated calorie intake during the intervention period.

After four weeks of resistance exercise, the change in muscle mass was significantly different between the two groups. The PSG showed significantly increased muscle mass, whereas the CON did not. Although the increase in muscle mass was relatively small (effect size, 0.56), considering that body weight was also significantly decreased, this increase in muscle mass could be meaningful. These results suggest that whey protein supplementation has an additional effect on resistance exercise-induced increases in muscle mass. Although the additional effects of protein supplementation during resistance exercise appear to be controversial, previous studies support our results. A recent meta-analysis reported that protein supplementation had a slight positive effect during resistance exercise [18]. A few studies have also reported a significant interaction between protein supplementation and changes in muscle mass, lean body mass, or fat-free mass [24]. However, these studies used dietary questionnaires to control for a normal diet. To the best of our knowledge, this study is the first to directly control normal diet. Therefore, our results indicate the effectiveness of whey protein supplementation.

Changes in body weight, body fat mass, and percent body fat were not significantly different between the two groups in this study. Similar results have been reported in previous studies. The most recent meta-analysis reported no significant effect of whey protein supplementation on body mass, body fat mass, or body fat percentage [25]. Considering that decreases in body fat mass and body fat percentage are well-known effects of resistance exercise [26], it is possible that the significant decreases in body composition in both groups in this study may be mainly due to resistance exercise. Taken together, there may be no additional effect of whey protein supplementation on resistance exercise-induced changes in body weight or body fat.

Interestingly, body weight significantly decreased after resistance exercise in both groups in this study. This could be mainly due to the significant decrease in body fat mass of approximately 20% in the PSG and 12% in the CON. Because the changes in muscle mass were much smaller than the changes in body fat mass, overall weight loss could have occurred. In fact, in the PSG, the change in body fat mass was −3.2 kg, while the change in muscle mass was +0.3 kg. In the CON, the change in body fat mass was −2.0 kg, while there were no changes in muscle mass. Another possibility for weight loss in this study may be that the actual calorie intake was lower than the estimated caloric intake (approximately 4.6% in the PSG and 4.4% in the CON). We attempted to accurately calculate the daily calorie intake required for each participant and provided the same number of calories. However, each person’s meal menu was diverse; therefore, it was difficult to match the exact calories in the food preparation process. Nevertheless, these unintentionally reduced calorie intakes could be considered to be within acceptable variability for a typical diet because even in previous studies with calorie restriction, the variation in calorie intake was approximately 6% to 10% [27,28]. Most weight loss programs reduce energy intake by 25–60% below the expected energy requirements, with the goal of losing an average of 5–8% of weight [29,30,31]. However, the possibility that these small calorie reductions could lead to weight loss cannot be ruled out. Lastly, participants’ daily calorie intake was estimated by multiplying their RMR by their activity factor [22]. Thus, it is also possible that energy expenditure during resistance exercise was underestimated, resulting in a negative energy balance, leading to weight loss in this study.

We measured isokinetic muscular function in parallel with muscle mass in this study because it is possible that muscular strength changes due to adaptations that occur within the muscle, regardless of muscle hypertrophy [32]. After 4 weeks of resistance exercise, there were significant differences between the two groups in selective muscular strength of the knee and shoulder. The PSG group showed significantly increased strength of both the dominant and non-dominant knees and shoulders, whereas the CON group did not. Although trunk strength did not change in either group, these results suggest that whey protein supplementation has substantial overall effects on the increases in muscular strength from resistance exercises. In previous studies examining the additional effects of protein supplementation combined with resistance exercise on muscular strength, most evaluated muscular strength using RM. Although the isokinetic muscular function test, which is considered the gold standard for muscular strength evaluation, can provide clearer evidence, studies using the isokinetic muscular function test for muscular strength are rare. Contrary to our results, previous studies using isokinetic muscle function tests have not reported the beneficial effects of protein supplementation [33,34]. However, it is possible that normal diets were not well controlled because these studies used dietary recall. Thus, the partial increase in the peak torque of the upper and lower extremities in the isokinetic muscular function test under the direct diet control condition in this study may indicate the actual effect of protein supplementation without the effect of diet.

Interestingly, in this study, muscular strength did not increase even after resistance exercise in the CON. This is probably because muscle hypertrophy did not occur or because the exercise period in this study was shorter than that in the previous studies. In fact, it has been reported that muscular strength gains can be induced by resistance exercise after at least six weeks [35].

The difference in muscular strength increases between the two groups was found only in the flexors for knees and extensors for shoulders. These results may be related to the type of exercise program. Among the exercises in our program, the high foot position leg press was suitable for increasing the strength of the knee flexors [36], whereas exercises such as weight-bearing squats and lunges may be insufficient for improving to improve the isokinetic strength of the knee extensors. Shoulder extensors seem to respond faster to exercise because their muscles are larger than the shoulder flexors [37]. In addition, seated and barbell rows among the exercise programs were suitable for increasing the muscular strength of the shoulder extensors.

Similar to the results for muscular strength, there were also significant differences between the two groups in selective muscular endurance of the knee and shoulder. The PSG showed significantly increased endurance of the dominant knee and shoulder, whereas the endurance of the CON did not change. Although there was no interaction between the groups, trunk endurance significantly increased in the PSG. Thus, these results suggest that whey protein supplementation may also have substantial overall effects on the increase in muscular endurance induced by resistance exercise. Although there are differences in the combinations of protein supplements, previous studies also support our findings. Jang et al. [38] reported that protein supplementation for 12 weeks combined with resistance exercise without normal dietary control produced a synergistic effect on muscular endurance. Our results also showed that whey protein supplementation had a partial additional effect on upper- and lower-body muscular endurance. Thus, it is believed that the actual effect of protein supplementation was revealed more clearly through the direct control of the normal diet.

Interestingly, differences in muscular endurance gains between the two groups were found only in the dominant extensors for both knees and shoulders. These results may also be related to our resistance exercise program. In this study, exercises related to knee joint muscular function were squats, lunges, and leg presses, which mainly used extensors. Knee joint extensors are continuously trained during lower-body exercises, which seems to improve muscular endurance. The major muscles involved in the extension of the glenohumeral joint are the latissimus dorsi and teres major [37]. As this muscle is larger than the anterior deltoid, which is the flexor of the glenohumeral joint, it must have been more affected by the increase in muscular endurance due to training. Thus, protein supplementation appears to be beneficial for the development of muscular endurance in the dominant extensors of the knee and shoulder by maximizing the positive effects of resistance exercise.

## 5. Conclusions

The results of this study indicate that whey protein supplementation promotes muscle mass increase and selective increases in muscular strength and endurance from resistance exercise. This suggests that whey protein supplementation is practically effective independently of the normal diet.

## Figures and Tables

**Table 1:** Changes in body composition after 4 weeks of resistance exercise.

Variables	PSG (*n* = 17)		*t*-Test ^a^		CON (*n* = 15)		*t*-Test ^a^		ANOVA ^b^
	Pre	Post	*p*	ES	Pre	Post	*p*	ES	*p*
Body weight (kg)	72.79 (2.42)	71.44 (2.77)	0.003	0.85	73.74 (4.24)	71.50 (3.89)	<0.001	1.32	0.134
Body fat mass (kg)	15.99 (4.57)	12.81 (5.43)	0.012	0.69	17.05 (4.04)	15.07 (3.24)	<0.001	1.38	0.345
Body fat percentage (%)	21.86 (5.72)	19.41 (5.88)	<0.001	2.09	22.98 (4.74)	20.99 (3.96)	<0.001	1.28	0.345

Values are presented as the mean (standard deviation). PSG, protein supplement group; CON, placebo group; ES, effect size (Cohen’s d). ^a^ Paired *t*-test; ^b^ Repeated-measures ANOVA.

**Table 2:** Changes in isokinetic muscular function for the trunk.

Variable		PSG (*n* = 17)		*t*-Test ^a^		CON (*n* = 15)		*t*-Test ^a^	ANOVA ^b^
		Pre	Post	*p*	ES ^c^	Pre	Post	*p*	*p*
Peak torque (N·m)	Ext	209.29 (44.63)	198.59 (29.70)	0.184		198.53 (43.36)	203.27 (27.06)	0.641	0.224
	Flx	181.94 (40.38)	186.24 (42.81)	0.646		188.67 (26.69)	177.93 (31.24)	0.201	0.232
Total work (J)	Ext	2518.65 (862.93)	2470.06 (804.57)	0.801		2073.13 (1245.06)	2189.40 (850.19)	0.598	0.568
	Flx	1891.53 (723.12)	2216.94 (709.56)	0.007	0.74	2148.67 (946.49)	2256.93 (677.77)	0.514	0.260

Values are presented as the mean (standard deviation). PSG, protein supplement group; CON, placebo group; Ext, extensors; Flx, flexors; ES, effect size; N·m, Newton-meter; J, joule. Assessments of peak torque and total work were performed at 60°/s and 180°/s respectively, during joint flexion-extension. ^a^ Paired *t*-test; ^b^ Repeated-measures ANOVA; ^c^ Cohen’s d.

## Data Availability

Not applicable.

## Appendix A

**Table A1:** Changes in isokinetic muscular function for the knee joint and shoulder joint.

Variable			PSG (*n* = 17)		*t*-Test ^a^		CON (*n* = 15)		*t*-Test ^a^		ANOVA ^b^	
			Pre	Post	*p*	ES ^c^	Pre	Post	*p*	ES ^c^	*p*	ES ^d^
Peak torque (N·m)												
Knee	Ext	D	145.06 (26.85)	154.29 (28.00)	0.167		155.73 (29.30)	152.33 (24.50)	0.558		0.154	
		ND	148.12 (21.35)	154.82 (27.48)	0.226		149.33 (29.60)	150.60 (21.91)	0.820		0.482	
	Flx	D	83.59 (17.37)	97.76 (17.58)	0.001	1.02	90.53 (18.60)	94.80 (12.42)	0.234		0.048	0.12
		ND	87.76 (14.87)	95.71 (13.55)	0.006	0.76	92.47 (18.85)	93.47 (14.66)	0.815		0.156	
Shoulder	Ext	D	65.35 (12.27)	71.18 (16.36)	0.004	0.83	66.60 (12.57)	67.80 (11.16)	0.212		0.028	0.15
		ND	62.65 (14.43)	69.71 (16.93)	0.001	0.93	64.80 (13.48)	64.73 (9.82)	0.975		0.015	0.18
	Flx	D	56.18 (9.04)	56.53 (5.98)	0.836		54.33 (6.77)	54.60 (6.10)	0.866		0.970	
		ND	52.94 (6.69)	54.59 (7.02)	0.217		54.67 (7.34)	51.93 (8.89)	0.149		0.052	
Total work (J)												
Knee	Ext	D	1680.88 (270.11)	1839.06 (202.34)	0.007	0.75	1879.47 (281.54)	1868.00 (247.70)	0.753		0.012	0.19
		ND	1805.53 (277.83)	1899.12 (229.67)	0.019	0.64	1656.47 (239.19)	1813.80 (233.85)	0.010	0.77	0.315	
	Flx	D	1160.53 (265.59)	1211.71 (219.39)	0.093		1084.13 (241.56)	1159.87 (209.67)	0.096		0.628	
		ND	1144.41 (210.80)	1193.24 (204.12)	0.223		1120.33 (232.62)	1098.73 (228.75)	0.642		0.243	
Shoulder	Ext	D	1074.35 (338.81)	1313.00 (310.71)	<0.001	1.17	1297.87 (286.84)	1371.60 (257.90)	0.057		0.013	0.19
		ND	1204.65 (446.45)	1278.82 (392.70)	0.154		1160.93 (387.74)	1302.00 (275.00)	0.027	0.64	0.381	
	Flx	D	1031.76 (269.16)	1085.24 (294.11)	0.161		1029.93 (382.95)	1062.47 (352.38)	0.524		0.732	
		ND	941.94 (232.09)	1020.65 (202.27)	0.108		1126.53 (296.83)	1077.00 (304.17)	0.280		0.055	

Values are presented as the mean (standard deviation). PSG, protein supplement group; CON, placebo group; Ext, extensors; Flx, flexors; D, dominant; ND, non-dominant; ES, effect size; N·m, newton-meter; J, joule. Assessments of peak torque and total work consisted of 60°/s and 180°/s respectively, during joint flexion-extension. ^a^ Paired *t*-test; ^b^ Repeated-measures ANOVA; ^c^ Cohen’s d; ^d^ Partial eta squared.
